# TRANSPLANTATION OF ADIPOSE-DERIVED MESENCHYMAL STEM CELLS IN REFRACTORY CROHN’S DISEASE: SYSTEMATIC REVIEW

**DOI:** 10.1590/0102-672020190001e1465

**Published:** 2019-12-20

**Authors:** Luana BERNARDI, Carlos Henrique Marques dos SANTOS, Verônica Assalin Zorgetto PINHEIRO, Rodrigo Juliano OLIVEIRA, Andreia Conceição Milan Brochado ANTONIOLLI-SILVA

**Affiliations:** 1 Postgraduate Program in Health and Development in the Center-West Region, Faculty of Medicine, Campo Grande, MS, Brazil;; 2 Stem Cell Study Center, Cell Therapy and Toxicological Genetics, Maria Aparecida Pedrossian University Hospital, Campo Grande, MS, Brazil;; 3 Coloproctology Department, Maria Aparecida Pedrossian University Hospital, Federal University of Mato Grosso do Sul, Campo Grande, MS, Brazil

**Keywords:** Crohn disease, Mesenchymal stem cell transplantation, Adipose tissue, Doença de Crohn, Transplante de células-tronco mesenquimais, Tecido adiposo

## Abstract

**Background:**

Crohn’s disease is a pathological condition that has different options of treatment, but there are patients who need other therapeutic approach, such as the use of adipose-derived mesenchymal stem cells.

**Aim:**

Systematic literature review to determine the different ways of adipose-derived mesenchymal stem cells administration in humans with luminal refractory and perianal fistulizing Crohn’s disease.

**Methods:**

It was conducted a search for articles (from 2008 to 2018) on PubMed and ScienceDirect databases using the keywords Crohn’s disease, fistulizing Crohn’s disease, luminal Crohn’s disease and transplantation of mesenchymal stem cells or mesenchymal stem cells or stromal cells. Thirteen publications were selected for analysis.

**Results:**

Only one study referred to the luminal Crohn´s disease. The number of cells administered was variable, occurring mainly through subcutaneous adipose tissue by liposuction. It could be highlighted the autologous transplant with exclusive infusion of mesenchymal stem cells. The procedures involved in pre-transplant were mainly curettage, setons placement and stitching with absorbable suture, and conducting tests and drug treatment for luminal Crohn´s disease. During transplant, the injection of mesenchymal stem cells across the fistula path during the transplant was mainly on the intestinal tract wall.

**Conclusion:**

Although the use of mesenchymal stem cells is promising, the transplant on the luminal region should be more investigated. The injection of mesenchymal stem cells, exclusively, is more explored when compared to treatment with other products. The preparation of the fistulizing tract and the location of cell transplantation involve standardized health care in most studies.

## RESUMO

**Racional:**

Há diferentes opções de tratamento para a doença de Crohn, porém, em alguns casos, há a necessidade de outras abordagens terapêuticas, como o uso de células-tronco mesenquimais derivadas do tecido adiposo.

**Objetivo:**

Revisar sistematicamente a literatura para determinar as diferentes formas de administração das células-tronco mesenquimais derivadas do tecido adiposo em seres humanos com doença de Crohn refratária luminal e fistulizante perianal.

**Método:**

Buscaram-se artigos publicados entre 2008 e 2018 nas bases de dados PubMed e ScienceDirect, pelos descritores: Crohn’s disease, fistulizing Crohns disease, luminal Crohns disease e transplantation of mesenchymal stem cells ou mesenchymal stem cell ou stromal cells. Treze artigos foram selecionados.

**Resultados:**

Somente um trabalho se referiu à doença luminal. A quantidade de células administradas foi variável, obtendo-se principalmente do tecido adiposo subcutâneo por lipoaspiração. Destacou-se o transplante autólogo com a infusão exclusiva de células-tronco mesenquimais. Os procedimentos realizados no pré-transplante foram principalmente o de curetagem, colocação de setons e suturas com fio absorvível, e de exames e tratamento medicamentoso para a doença luminal. No transplante, ocorreu a injeção das células por todo o trajeto fistuloso, principalmente nas paredes do trato.

**Conclusão:**

Embora o uso de células-tronco mesenquimais seja promissor, o transplante na região luminal deve ser mais investigado. A injeção exclusiva de células-tronco mesenquimais é mais explorada quando comparada ao tratamento conjunto com outros produtos. A forma de preparo do trato fistuloso e o local de transplante envolvem cuidados médicos padronizados na maioria dos estudos.

## INTRODUCTION

Crohn’s disease (CD) is an inflammatory bowel disease (IBD) that compromises the person’s health because of its chronic and relapsing condition on the gastrointestinal tract^1^. Among the most common complications of this disease, there is the perianal fistulas, which form when there is an abnormal connection between the intestinal wall and other organ or the skin^7^ ^,^ ^23^. Its prevalence varies geographically, with Brazil the only country from Latin America considered with high incidence of cases^2^ ^,^ ^32^. Besides that, perianal fistulas can affect about 28% of patients within 20 years after diagnosis^14^. 

Although there are different options for control of the clinical condition, there are refractory patients to treatment, and requiring other options to control gastrointestinal inflammation or to promote the healing process. The use of adipose-derived mesenchymal stem cells (MSC) has shown benefits, being capable to enhance the regeneration and repair of damaged tissues^25^. Its effectiveness is due primarily to the immunomodulatory and anti-inflammatory potencial^3^, in addition to the fact that most of the treatments do not declare the occurrence of adverse reactions associated with the transplant^10^. Besides that, the MSCs have high proliferation and differentiation capacity. Although they can be isolated from different tissues, getting through the adipose tissue is considered the largest source, by giving low morbidity and discomfort to the patient, as they can be obtained in large quantities and through easy isolation techniques^22^. 

It is still controversial in the literature the most appropriate technique for the MSCs transplant. As an example, there are a variety of agents administered in perianal fistulas, like fibrin glue, plugs, hyperosmolar glucose solution and doxycycline, among others^20^ some inserted along with the stem cells. In addition, it is noticed a disparity about the best body location of adipose tissue to obtain the MSCs, the amount of these cells being administered in therapy, the type of transplant, among other controversial factors. 

Thus, the aim of this study was to realize a sistematic review of the literature to determine the different ways of adipose-derived MSC administration in humans with luminal refractory and perianal fistulizing CD.

## METHODS

Articles based on PubMed and ScienceDirect databases and published on the last 10 years (from january 2008 to december 2018) were evaluated. The search for abstracts was performed using the Boolean operator [AND] between the following keywords: Crohn’s disease, fistulizing Crohn’s disease, luminal Crohn’s disease and transplantation of mesenchymal stem cells or mesenchymal stem cell or stromal cells. 

Studies were selected through the following inclusion criteria: a) favorable/unfavorable results from the process of intervention on perianal fistula or intestinal tract lumen/mucosa; b) sample composed of individuals with refractory Crohn’s disease; c) published between 2008 and 2018 and; d) studies related to treatments with adipose-derived MSCs. It was adopted the following exclusion criteria: a) reviews, editorials, commentaries or letters; b) without complete methodological description (objectives, methods and results); c) studies not regarding the treatment with adipose-derived MSCs; d) studies of fistulas in non-perianal localization; e) studies including patients with other IBD than CD and; f) studies not regarding patients with refractory CD. It was also considered as an exclusion criterion duplicated articles, which were manually deleted.

According to the eligibility criteria, two authors (LB and ACMBAS) selected the studies independently in two stages: evaluating the title and summary and, subsequently, by reading the full text. Disagreements were resolved by consensus.

### Intervention with MSCs in luminal and fistulizing perianal CD

**FIGURE 1:**
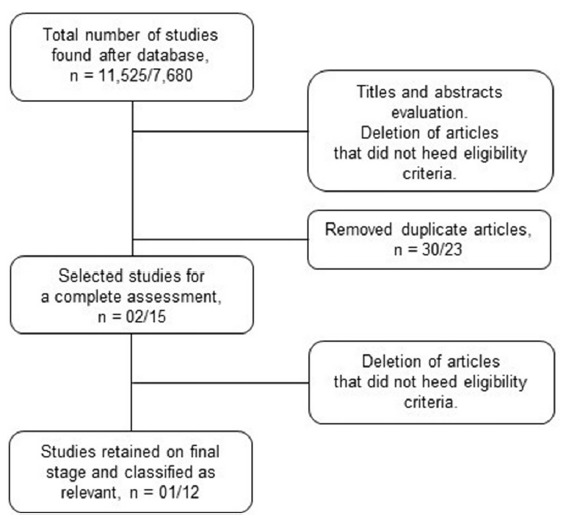
Flowchart of the selection process-related articles with the themes “luminal Crohn’s disease” and “perianal fistulizing Crohn’s disease”, respectively

**FIGURE 2:**
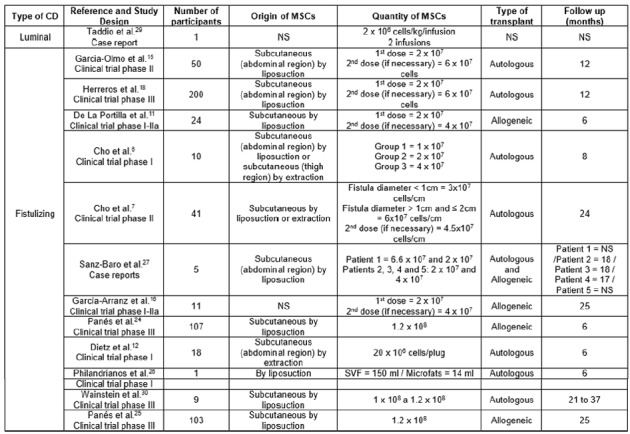
Characteristics of the mesenchymal stem cells transplantation studies in perianal fistulas and in the intestinal lumen of patients with refractory Crohn’s disease

**FIGURE 3:**
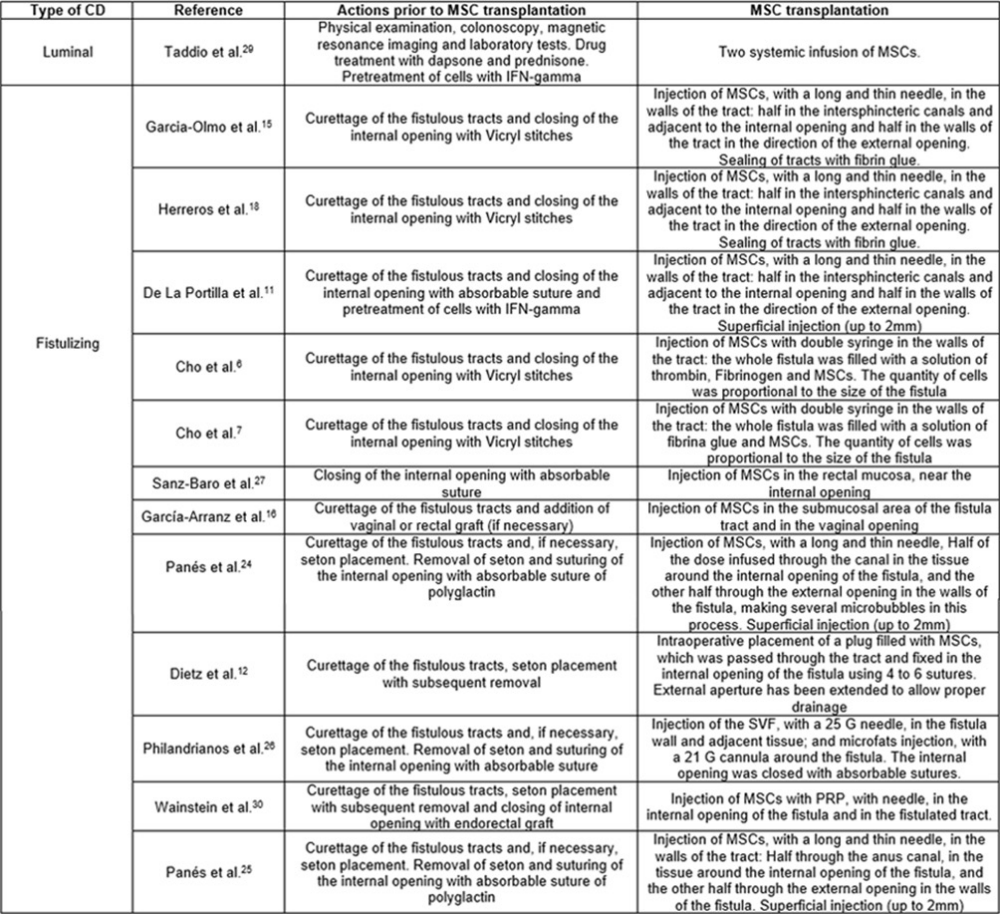
Assessment of the mesenchymal stem cells transplantation in perianal fistulas and in the intestinal lumen of patients with refractory Crohn’s disease.

For both subthemes were found, respectively, a total of 11,525/7.680 articles (PubMed: 982/522 and ScienceDirect: 10,543/7,158). The descriptors used were: Crohn’s disease (Mesh), luminal Crohn’s disease (TIAB), mesenchymal stem cells transplantation (Mesh), mesenchymal stem cell (Mesh) and stromal cells (Mesh) for ‘luminal Crohn’s disease’ and Crohn’s disease (Mesh), fistulizing Crohn’s disease (TIAB), mesenchymal stem cells transplantation (Mesh), mesenchymal stem cell (Mesh) and stromal cells (Mesh) for ‘fistulizing perianal Crohn’s disease’. The exclusion was as follows, respectively: reviews, editorials, commentaries or letters (1,813/1,915); incomplete methodoloy (12/9); intervention without the use of adipose-derived MSCs (110/134); studies using animal models (98/86); studies not regarding patients with refractory CD (213/323); have not been published in the last 10 years (9,247/5,175); and duplicated articles (30/23). After the inclusion and exclusion criteria were applied, it has remained, respectively: 02/15 articles (PubMed: 01/10 and ScienceDirect: 01/09), which were read in full. Then, for the subtheme “luminal Crohn’s disease” was excluded one article that was not in accordance with the criterion: studies regarding the treatment with adipose-derived MSCs. For the subtheme “perianal fistulizing Crohn’s disease” we excluded three more studies that were not consistent with the criterion: sample composed of individuals with refractory Crohn’s disease. In this way, a total of 01/12 studies remained (Figure 1), respectively, Which have been tabulated, with the description of the following items: type of CD, reference and study design, number of participants, source of MSCs, quantity of MSCs, type of transplant, follow up (Figure 2) and; previous action for MSCs transplantation (Figure 3).

CD=Crohn’s disease; MSC=mesenchymal stem cell; SVF=stromal vascular fraction; NS=non-specified

CD=Crohn’s disease; MSC=mesenchymal stem cell; NS=non-specified; INF= interferon; SVF=stromal vascular fraction; PRP=platelet-rich plasma

## RESULTS

The main features found in the 13 included studies are detailed in Figures 2 and 3. Among these, one study is about luminal CD^28^ and the other 12 about perianal fistulizing CD^6^ ^,^ ^7^ ^,^ ^11^ ^,^ ^12^ ^,^ ^15^ ^,^ ^16^ ^,^ ^18^ ^,^ ^24^ ^,^ ^25^ ^,^ ^26^ ^,^ ^27^ ^,^ ^30^. 

### Review of the central objective

#### Forms of administering the MSCs in the fistulized perianal tract and in the intestinal lumen of patients with refractory CD

Regarding the dose of MSCs, the number of transplanted cells ranged from 2x10^6^ to 1,2 x10^8^ cells^24^ ^,^ ^25^ ^,^ ^29^, and two studies infused a cell volume proportional to the size of the fistula^6^ ^,^ ^7^ and other studies the amount of cells obtained from culture, regardless of the size of the fistula. With respect to the infusion of cells with other substances, in two studies the MSCs underwent a pre-treatment with interferon gamma (*IFN*-γ)^11^ ^,^ ^29^ and only five had the MSCs injected with a mixed solution of thrombin and fibrinogen^6^, platelet-rich plasma^30^ or fibrin glue^6^ ^,^ ^7^. 

The obtainance of adipose tissue was held from similar sources. Ten studies made use of subcutaneous tissue^6,7,11,12,15,18,24,25,27,30^ five of which from the abdominal region^6,12,15,18,27^ , one from the thigh region^6^ and others have not reported^7^ ^,^ ^11^ ^,^ ^16^ ^,^ ^24^ ^,^ ^25^ ^,^ ^26^ ^,^ ^29^ ^,^ ^30^. Ten studies obtained the adipose tissue through liposuction technique^6^ ^,^ ^7^ ^,^ ^11^ ^,^ ^15^ ^,^ ^18^ ^,^ ^27^ ^,^ ^24^ ^,^ ^26^ ^,^ ^29^ ^,^ ^30^, and three also obtained through the extraction technique for obtaining microfragmented adipose tissue^6^ ^,^ ^7^ ^,^ ^12^. Two studies did not report the local nor the way of obtaining the adipose tissue^16,29^ and one reported only as obtained tissue^26^. 

The comparison of the transplantation method of the MSCs was possible only for perianal fistulizing CD. Five studies conducted allogeneic transplantation^11^ ^,^ ^16^ ^,^ ^24^ ^,^ ^25^ ^,^ ^27^ and eight autologous transplantation^6,7,12,15,18,26,27,30^ and one^27^ held one or the other type of transplant in the course of the investigation, depending on the patient. For the perianal fistulizing CD, transplantation occurred by systemic infusion^29^. 

Regarding the preparation of fistulous tracts for cellular infusion, 11 studies described the performance of curettage procedures^6^ ^,^ ^7^ ^,^ ^11^ ^,^ ^12^ ^,^ ^15^ ^,^ ^16^ ^,^ ^18^ ^,^ ^24^ ^,^ ^25^ ^,^ ^26^ ^,^ ^30^, one the drainage procedure^30^, ten described closure of internal fissures with absorbable suture^6^ ^,^ ^7^ ^,^ ^11^ ^,^ ^12^ ^,^ ^15^ ^,^ ^18^ ^,^ ^24^ ^,^ ^27^ ^,^ ^25^ ^,^ ^26^, five with the description of prior placement of seton and subsequent withdrawal before the cell infusion^12^ ^,^ ^24^ ^,^ ^25^ ^,^ ^26^ ^,^ ^30^, and two of them described the use of grafts (vaginal and/or rectal) to close the fissures^16^ ^,^ ^30^. 

On the use of instruments for the infusion of cells, a study performed the procedure through the introduction of plugs with MSCs^12^, and the others did the application directly in the fistula with a surgical needle^6^ ^,^ ^7^ ^,^ ^11^ ^,^ ^15^ ^,^ ^16^ ^,^ ^18^ ^,^ ^24^ ^,^ ^25^ ^,^ ^26^ ^,^ ^30^. The majority performed the procedure for the extraction of MSCs in a laboratory environment for subsequent injection in the fistulas. However, one study^26^ performed the injection of the stromal vascular fraction derived from adipose tissue and the microfats graft in the fistula.

The description of the transplant procedure proved to be homogeneous in most studies. The injection of MSCs throughout the fistulous pathway, mainly in the walls of the tract, was present^6^ ^,^ ^7^ ^,^ ^11^ ^,^ ^15^ ^,^ ^18^ ^,^ ^24^ ^,^ ^25^ ^,^ ^26^, highlighting a superficial injection of up to 2 mm in some studies^11^ ^,^ ^24^ ^,^ ^25^. The closure of the fistula after the cell injection was cited by few authors, by means of fibrin glue^15,18^ or absorbable suture^26^. 

## DISCUSSION

Characteristics of the mesenchymal stem cells transplantation studies in the fistulous tract and in the intestinal lumen 

This study shows the growing interest in adipose-derived MSCs transplantation by different techniques, having as a common goal to potentiate the treatment of patients with refractory CD that have or not perianal fistulas. The results indicate that the literature lacks when this approach turns to patients with luminal CD. In a recent literature review^22^, it is observed that systemic infusions of MSCs to treat luminal CD have been experienced, having the bone marrow as the preferred source of these cells. In the paper of Bor and coworkers^3^, only four studies aiming to treat luminal CD with systemic infusion of MSCs were raised. Among these, three had as source the bone marrow and one, the umbilical cord. These facts contribute to justify the inclusion of only one paper in this review, suggesting that there should be more attempts to make use of adipose tissue as a source of MSCs.

The quantity of transplanted cells has varied among the studies. This may be justified by different techniques of isolation and culture in vitro, which causes different protocols leading to different influences on the growth of MSCs. Thus, the use of various culture media, cell density and hypoxia, use of flasks of different sizes, as well as the addition of growth factors during cultivation, besides the characteristics of the donor (age, gender, ethnicity, body mass index and medical history), type of adipose tissue (yellow/brown) and localization (subcutaneous/visceral fat)^4^, may interfere on the final result. Some studies have described the use of supplements that assist the cell expansion process, such as the fetal bovine^6^ ^,^ ^12^ ^,^ ^15^ ^,^ ^16^ ^,^ ^24^ ^,^ ^25^, human albumin^11^ and fibroblast growth factor^6^ ^,^ ^7^. However, the optimum dosage to increase cure rates remains an important issue to be defined, along with the ideal time for repeated injections and the optimization of treatment protocols^20^.

Although the analysis of the isolation and expansion protocols of stem cells is not the focus of this review, its standardization can guarantee that MSC-based therapies become generalized approaches. About this aspect, there is a slight disparity between the place where adipose tissue is obtained in the articles analyzed. Among those who made the information available, the adipose tissue of the subcutaneous region was the most required, mainly by the liposuction technique. Some tissues are richer in MSCs than others, what makes them more used^22^. In fact, the subcutaneous adipose tissue is considered an easily and accesible source of large amounts by minimally invasive procedures (aspiration or liposuction^4^. Liposuction is considered low-invasive, inexpensive and provides an adequate number of cells even in small amounts^22^. Besides that, the abdomen is the most common site of adipose tissue collection, followed by the trochanteric and inner regions of the thighs and knees^19^, which is also in agreement with this present review.

Obtaining sufficient quantities of MSCs in vitro, the clinical application of these in the patient may occur in autologous or allogenic way^22^. It is worth mentioning that the ability to inhibit immune responses^3^ also confers on MSC protection against transplant rejection. However, the risk of allogeneic-derived MSC being rejected by immunocompetent patients is greater^5^. In the present review, most of the selected studies opted for autologous transplantation^8^. This type of transplant is considered the best option, because the chances of stimulating an immunological response is practically none^22^. Another point to consider is that the survival of autologous MSC in the body is superior when compared to a material from a donor^5^. 

Regarding the product that can be transplanted in the patient with refractory CD, different alternatives were used in the articles, such as the exclusive use of MSC^6^ ^,^ ^7^ ^,^ ^11^ ^,^ ^12^ ^,^ ^15^ ^,^ ^16^ ^,^ ^18^ ^,^ ^24^ ^,^ ^25^ ^,^ ^27^ ^,^ ^30^, the stromal vascular fraction (SVF) and the microfats^26^. The mechanism of tissue regeneration due the SVF and the graft of small fat particles (microfats) has been investigated^9^. The SVF consist of a heterogeneous populations of cells, including the MSCs, but with a variable presence among the patients (≈3% is composed of MSC)^17^. According to Salgado et al.^28^, this fraction is capable to promote angiogenesis, wound healing and stem cell differentiation, what could be due to its paracrine effects of the cells. Besides that, the microfats transplantation is associated with correction of scars and wound healing, for example^9^. However, Philandrianos et al.^26^, even obtaining good results, have presented the first literature report that made a combined use of both products to treat perianal fistula on CD, which raises the need for more studies by comparing the effectiveness of the three treatments. 

A case series^15^ verified the treatment of enterocutaneous perianal fistula with adipose-derived MSCs and with the SVF. Patients who have received the in vitro-expanded MSCs had a greater cure obtainance when compared with the other group. The use of SVF can promote good results; however, due its heterogeneous cell population, it is mandatory to understand its mechanism of action before introducing it in the regular clinical practice, issue that has been under a wide discussion when it comes to the transplant of stem cells. On the other hand, Philandrianos et al.^26^ referred that the perspective of cost-effectiveness of treatment should be taken into consideration, since obtaining the SVF requires only a few hours, and not weeks as is the case of stem cells culture, allowing liposuction and reinjection on the same day. Thus, it is understood that new therapeutic strategies have been investigated for the treatment of patients with perianal fistula associated with refractory CD, But that the feasibility of these procedures should be tested in larger groups of patients, as well as the comparison of their efficacy.

### Assessment of the mesenchymal stem cells transplantation in perianal fistulas and in the intestinal lumen 

The transplant itself requires the preparation of the region that will receive the product (MSC, SVF or microfats). The studies, which made information available, reported similar care to the participants before performing the transplant (Figure 3). Three stages showed patterns, such as curettage of the fistulated epithelium tracts, placement and posterior removal of setons and suturing of the tracts with absorbable sutures. The curettage process promotes the exposure of MSC to a healthy tissue, being considered an effective and recognized mechanism among the treatments. In this process, the placement of setons can be useful by avoiding the formation of abscesses, since it maintains clean the path of the fistula, but it can lead to the formation of a fibrotic tissue in some cases which would decrease the local blood supply for the injection of cells. Moreover, among the studies that made use of setons, the moment it was placed ranged from 1-2^24,25,26^ weeks up to 4-6 weeks^30^ before the suture of the fistula and the cell injection, with removal immediately before these steps. Among the absorbable sutures specified, it was cited Vicryl^®^ and polyglactin stitches. Regardless not having any restriction about the type of suture material, the use of polyglactin suture was the most indicated by the literature^17^.

During the cell injection process, the use of fibrin glue was cited, both in combination with MSCs^6^ ^,^ ^7^ ^,^ ^18^ and to close fistula after cell infusion^15^. Kotze et al.^20^ described that the surgical management of removal of setons and the curettage process can be considered the best approach before injecting the glue. The glue is not considered a cytotoxic product, is capable of stimulating the cellular adhesion and growth, being studied as a vehicle for the MSCs in regenerative medicine^31^. In this respect, the interest in the application of fibrin glue, especially in conjunction treatment with stem cells in perianal fistula, is associated with the healing capacity, both by angiogenic action of the fibrin matrix^13^ and by MSCs, as by capacity of secretion of cellular growth and differentiation factors^8^. However, its application in combination with MSCs must be careful, since there is no sufficient scientific evidence for its recommendation, and does not yet exist a protocol defined for its use^17^. 

Several biological therapies for treatment of refractory CD have been explored and have been identified in the present study. It can be mentioned the use of tissue grafts^16^, plugs filled with cells^12^ and the application of platelet-rich plasma^30^. The use of these therapies in conjunction with the MSCs appears in the interest of improving the effectiveness of treatment, reducing the risk of incontinence in patients. Thus, there is a need for studies that compare the effectiveness of different modes of administration of the MSCs, as well as direct and exclusive administration of the cells is superior to use in conjunction with biological therapies. However, it is known that these can be useful also to maintain the MSCs in place administered by a longer time, which may contribute to an increase in the rates of cure^21^. 

Although the MSC-based treatment can increase the regenerative capacity of the tissue, improving surgical results, there is still no clear surgical guidelines for the application of stem cell therapy^17^. In this review, the administration site of these cells proved standard on most studies, with the infusion on both internal and external holes of fistula, giving special attention to the injection on the walls of the fistulous tract^6^ ^,^ ^7^ ^,^ ^11^ ^,^ ^15^ ^,^ ^18^ ^,^ ^24^ ^,^ ^25^ ^,^ ^30^. These techniques are in accordance with the recent published step by step protocol on the treatment of perianal fistula with MSCs^17^. In this, the authors add that the cells should not be injected in contact with the lumen of the fistula, or away from the walls of the tract, once they exert local effect and can be eliminated with post-operative secretions.

## CONCLUSION

We can conclude that the use of adipose-derived MSCs is promising featured mainly for autologous transplantation. However, the transplant in the luminal region should be more investigated. The exclusive injection of MSCs in perianal fistula is best exploited when compared to treatment together with other products, which should be used with caution and present standard techniques to be used in clinical studies. Between the transplantation of MSCs or the SVF, the latter has been studied, but without enough evidence if it performs the same effective action about the healing process of perianal fistula. On the other hand, the form of preparation of the fistulated region, as well as the location of the cell transplant, was standard among most authors, demonstrating that the studies were following similar medical care.

## References

[1] Baumgart DC, Sandborn WJ (2012). Crohn&apos;s disease. Lancet.

[2] Behzadi P, Behzadi E, Ranjbar R (2015). The Incidence and Prevalence of Crohn&apos;s Disease in
Global Scale. SOJ Immunol.

[3] Bor R, Fábián A, Farkas K, Molnár T, Szepes Z (2018). Human mesenchymal stem cell therapy in the management of luminal
and perianal fistulizing Crohn&apos;s disease - review of pathomechanism
and existing clinical data. Expert Opin Biol Ther.

[4] Baer PC, Geiger H (2012). Adipose-Derived Mesenchymal Stromal/StemCells Tissue
Localization, Characterization, and Heterogeneity. Stem Cells Int.

[5] Barkholt L, Flory E, Jekerle V, Lucas-Samuel S, Ahnert P, Bisset L, Büscher D, Fibbe W, Foussat A, Kwa M (2013). Risk of tumorigenicity in mesenchymal stromal cell-based
therapies-bridging scientific observations and regulatory
viewpoints. Cytotherapy.

[6] Cho YB, Lee WY, Park KJ, Kim M, Yoo HW, Yu CS (2013). Autologous adipose tissue-derived stem cells for the treatment of
Crohn&apos;s fistula a phase I clinical study. Cell Transplant.

[7] Cho YB, Park KJ, Yoon SN, Song KH, Kim DS, Jung SH (2015). Long-Term Results of Adipose-Derived Stem Cell Therapy for the
Treatment of Crohn&apos;s Fistula. Stem Cells Transl Med.

[8] Cherubino M, Rubin JP, Miljkovic N, Kelmendi-Doko A, Marra KG (2011). Adipose-derived stem cells for wound healing
applications. Ann Plast Surg.

[9] Cohen N, Shani O, Raz Y, Sharon Y, Hoffman D, Abramovitz L (2017). Fibroblasts drive an immunosuppressive and growth-promoting
microenvironment in breast cancer via secretion of Chitinase 3-like
1. Oncogene.

[10] Dave M, Mehta K, Luther J, Baruah A, Dietz AB, Faubion WA JR (2015). Mesenchymal Stem Cell Therapy for Inflammatory Bowel Disease A
Systematic Review and Meta-analysis. Inflammatory Bowel Disease.

[11] De la Portilla F, Alba F, García-Olmo D, Herrerías JM, González FX, Galindo A (2013). Expanded allogeneic adipose-derived stem cells (eASCs) for the
treatment of complex perianal fistula in Crohn&apos;s disease results
from a multicenter phase I/IIa clinical trial. Int J Colorectal Dis.

[12] Dietz AB, Dozois EJ, Fletcher JG, Butler GW, Radel D, Lightner AL (2017). Autologous mesenchymal stem cells, applied in a bioabsorbable
matrix, for treatment of perianal fistulas in patients with Crohn&apos;s
disease. Gastroenterology.

[13] Dvorak HF, Harvey VS, Estrella P, Brown LF, McDonagh J, Dvorak AM (1987). Fibrin containing gels induce angiogenesis Implications for tumor
stroma generation and wound healing. Lab Invest.

[14] Eglinton TW, Barclay ML, Gearry RB, Frizelle FA (2012). The spectrum of perianal Crohn&apos;s disease in a
population-based cohort. Dis Colon Rectum.

[15] Garcia-Olmo D, Herreros D, Pascual I, Pascual JA, Del-Valle E, Zorrilla J, De-La-Quintana P, Garcia-Arranz M, Pascual M (2009). Expanded adipose-derived stem cells for the treatment of complex
perianal fistula a phase II clinical trial. Dis Colon Rectum.

[16] García-Arranz M, Herreros MD, González-Gómez C, De La Quintana P, Guadalajara H, Georgiev-Hristov T (2016). Treatment of Crohn&apos;s-Related Rectovaginal Fistula With
Allogeneic Expanded-Adipose Derived Stem Cells A Phase I-IIa Clinical
Trial. Stem Cells Transl Med.

[17] Georgiev-Hristov T, Guadalajara H, Herreros MD, Lightner AL, Dozois EJ, García-Arranz M (2018). A Step-By-Step Surgical Protocol for the Treatment of Perianal
Fistula with Adipose-Derived Mesenchymal Stem Cells. J Gastrointest Surg.

[18] Herreros MD, Garcia-Arranz M, Guadalajara H, De-La-Quintana P, Garcia-Olmo D (2012). Autologous expanded adipose-derived stem cells for the treatment
of complex cryptoglandular perianal fistulas a phase III randomized clinical
trial (FATT 1: fistula advanced therapy trial 1) and long-term
evaluation. Dis Colon Rectum.

[19] Hamza A, Lohsiriwat V, Rietjens M (2013). Lipofilling in breast cancer surgery. Gland Surg.

[20] Kotze PG, Shen B, Lightner A, Yamamoto T, Spinelli A, Ghosh S (2018). Modern management of perianal fistulas in Crohn&apos;s
disease future directions. Gut.

[21] Lightner AL, Wang Z, Zubair AC, Dozois EJ (2018). A Systematic Review and Meta-Analysis of Mesenchymal Stem Cell
Injections for the Treatment of Perianal Crohn&apos;s Disease Progress
Made and Future Directions. Dis Colon Rectum.

[22] Mishra T, Sarswat A, Mishra K, Srivastava A (2017). Inflammatory bowel diseases current therapeutic approaches and
potential of using stem cells. J Stem Cell Res Ther.

[23] Passos MAT, Chaves FC, Chaves-Junior N (2018). The importance of colonoscopy in inflammatory bowel
diseases. ABCD, arq. bras. cir. dig.,.

[24] Panés J, García-Olmo D, Van Assche G, Colombel JF, Reinisch W, Baumgart DC (2016). ADMIRE CD Study Group Collaborators Expanded allogeneic
adiposederived mesenchymal stem cells (Cx601) for complex perianal fistulas
in Crohn&apos;s disease: a phase 3 randomised, double-blind controlled
trial. Lancet.

[25] Panés J, García-Olmo D, Van Assche G, Colombel JF, Reinisch W, Baumgart DC (2018). Long-term Efficacy and Safety of Stem Cell Therapy (Cx601) for
Complex Perianal Fistulas in Patients With Crohn&apos;s
Disease. Gastroenterology.

[26] Philandrianos C, Serrero M, Grimaud F, Magalon J, Visée C, Velier M (2018). First clinical case report of local microinjection of autologous
fat and adipose-derived stromal vascular fraction for perianal fistula in
Crohn&apos;s disease. Stem Cell Res Ther.

[27] Sanz-Baro R, García-Arranz M, Guadalajara H, De La Quintana P, Herreros MD, García-Olmo D (2015). First-in-Human Case Study Pregnancy in Women With
Crohn&apos;s Perianal Fistula Treated With Adipose-Derived Stem Cells: A
Safety Study. Stem Cells Transl Med.

[28] Salgado AJ, Reis RL, Sousa N (2010). adipose tissue derived stem cells secretome: soluble factors and
their roles in regenerative medicine. Curr Stem Cell Res Ther.

[29] Taddio A, Tommasini A, Valencic E, Biagi E, Decorti G, De Iudicibus S (2015). Failure of interferon- pre-treated mesenchymal stem cell
treatment in a patient with crohn&apos;s disease. World J Gastroenterol.

[30] Wainstein C, Quera R, Fluxá D, Kronberg U, Conejero A, López-Köstner F (2018). Stem cell therapy in refractory perineal Crohn's disease:
long-term follow-up. Colorectal Dis.

[31] Wu Xiuwen, Ren Jianan, Jieshou Li (2012). Fibrin glue as the cell-delivery vehicle for mesenchymal stromal
cells in regenerative medicine. Cytotherapy.

[32] Xu M, Zhu P, Wang H, Yang B, Chen H, Zeng L (2019). Analysis of the clinical characteristics of perianal fistulising
crohn's disease in a single center. ABCD, arq. bras. cir. dig.

